# Seroprevalence of *Toxoplasma gondii* and associated risk factors among pregnant women in Jimma town, Southwestern Ethiopia

**DOI:** 10.1186/1471-2334-12-337

**Published:** 2012-12-05

**Authors:** Endalew Zemene, Delenasaw Yewhalaw, Solomon Abera, Tariku Belay, Abdi Samuel, Ahmed Zeynudin

**Affiliations:** 1 Department of Medical Laboratory Sciences and Pathology, College of Public Health and Medical Sciences, Jimma University, Jimma, Ethiopia; 2 Department of Biology, College of Natural Sciences, Jimma University, Jimma, Ethiopia; 3 Program of Medical Laboratory Science, Department of Medical Sciences, College of Medical and Health Sciences, Wollega University, Nekemte, Ethiopia

**Keywords:** Seroprevalence, Pregnant women, *T. gondii*, Ethiopia

## Abstract

**Background:**

Toxoplasmosis is a common parasitic infection caused by an obligate intracellular protozoan, *Toxoplasma gondii*. If primary toxoplasmosis occurs during pregnancy about one third of the cases could lead to congenital toxoplasmosis, with subsequent pathological effects. This study aimed at determining the seroprevalence of *T. gondii* among pregnant women in Jimma town, Southwest Ethiopia.

**Methods:**

A community based cross-sectional study was conducted to assess the seroprevalence and associated factors in pregnant women from August to September, 2011. A total of 201 study participants were included in this study. Data on socio-demographic and predisposing factors were collected from each study participant. Moreover, venous blood specimens were collected following Standard Operating Procedures. All the collected specimens were tested for IgM and IgG anti-*T. gondii* antibodies by enzyme-linked immunosorbent assay (ELISA).

**Results:**

The overall seroprevalence of *T. gondii* in the study area was 83.6%. One hundred and sixty three (81.1%) of the pregnant women were IgG seropositive, five (2.5%) were IgM seropositive. Three of the 5 pregnant women were positive for both IgG and IgM. Presence of domestic cat at home showed significant association with anti-*T. gondii* seropositivity (OR = 5.82, 95% CI: 1.61- 20.99; p < 0.05).

**Conclusion:**

The seroprevalence of *T. gondii* antibodies was high among the pregnant women. Pregnant women having domestic cat at their home were at higher risk of *T. gondii* infection. Hence, health education and awareness on the disease and its transmission to women of reproductive age group in general and pregnant women in particular should be created during antenatal follow up to reduce the risk of *T. gondii* infection in pregnant women.

## Background

Toxoplasmosis is a disease caused by an obligate intracellular protozoan parasite *Toxoplasma gondii*. It is commonly transmitted to humans by accidental ingestion of oocyst stage of the parasite after cleaning an infected cat's litter box. Other routes of transmission include accidental ingestion of the parasite in contaminated soil and drinking water, and consumption of infected raw meat. It can also be transmitted congenitally during pregnancy [1].

Generally, it is estimated that about one third of the World’s population is infected with *T. gondii*. High prevalence of the infection have been reported among pregnant women and women of childbearing age from different foci in Latin America, parts of Eastern/Central Europe, the Middle East, parts of south-east Asia and Africa [2]. However, the prevalence of *T. gondii* in pregnant women in China was less than 10% [3]. In Africa, overall seroprevalence rate as high as 92.5% has been reported [4]. Most pregnant women infected with *T. gondii* are chronically infected while few acquire the infection during pregnancy [5]. Pregnant women with acute infection during pregnancy are at risk of congenitally transmitting the infection to the fetus.

Congenital transmission as a result of primary infection during pregnancy is higher if the infection is acquired during the third trimester of pregnancy and is lower if the infection occurs during the first trimester. But, congenital infection occurring during the first trimester may result in a higher risk of tragic outcomes, which may include abortion [6], than the infection at the third trimester [7].

In spite of presence of stray cats and suitable climatic conditions favoring survival of the parasite in the study area, to our knowledge, there is no documented data on the epidemiology of *Toxoplasma* infection in the study area. Absence of documented data initiated us to undertake this study for evidence-based decision to support prevention and control of the disease. Besides this, serological screening of pregnant women for *T. gondii* is not practiced yet as an antenatal examination in health facilities in Ethiopia. Therefore, this study is aimed at determining seroprevalence of *T. gondii* and assessing associated factors among pregnant women in Jimma town.

## Methods

### Study area

The study was conducted in Jimma town, located 350 Kms southwest of the capital Addis Ababa. The town is divided into 13 *kebeles* (smallest administrative units in Ethiopia). According to the 2007 Central Statistical Agency census report [8] the projected total population of the town is 134, 040, females constituting 49.7%. The town is generally characterized by warm climate.

### Study design and sample size determination

A community based cross-sectional study was conducted from August to September 2011. The sample size was calculated using Epi Info (CDC, Atlanta, U.S.A., 2005) 6.04 statistical package. Sample size was calculated assuming the expected frequency of disease among unexposed group is 60% [9] and among population exposed 92.5% [4], 95% confidence level and 80% power, which gave us sample size of 64. After multiplying it by three for design effect and adding 10% for the anticipated non-response rate, the final sample size was calculated to be 211.

A multistage sampling technique was employed to select study participants. First, five kebeles were selected from the 13 kebeles of the town by lottery method. Then, the calculated sample size was allocated to the five selected kebeles proportional to the total number of pregnant women residing in each kebele. Finally, pregnant women in any of the three trimesters were selected by systematic sampling. Trained nurses, conversant of the local language interviewed the study participants about socio-demographic characteristics and associated predisposing factors using pretested semi-structured questionnaire. The questionnaire was first prepared in English (Additional file 1) and then translated to the local language (Afan Oromo). Moreover, venous blood specimens were collected from each study participant by experienced laboratory technologists following standard operating procedures.

### Specimen collection and laboratory processing

About 2ml of venous blood was collected by needle and syringe technique aseptically from each of the study participants. The blood samples were then transported to parasitology laboratory of the department of Medical Laboratory Sciences and Pathology. Then serum was separated from the whole blood by centrifugation at 3000 rpm for 5 min. Separated serum was labeled and kept at −20°C until use. Finally, it was tested for anti-*T. gondii* IgG and IgM antibodies using ELISA test kit (Human Gesellschaft für Biochemica und Diagnostica mbH*,* Germany) following the manufacturer’s instruction.

### Data analysis

Data collected were checked for completeness and consistency and the data were entered in to a computer and analyzed using SPSS version 16.0 software package. Bivariate and multivariate logistic regressions were used for the analysis. P-values less than 0.05 were considered statistically significant in the analysis.

### Ethical considerations

Ethical clearance was obtained from Jimma University Research and Ethics Review Committee and permission was obtained from Jimma Zone Health Bureau. Informed written consent was sought from each pregnant woman prior to involvement in the study. Information collected from each study participant was kept confidential and venous blood specimens collected were preserved anonymously.

## Results

### Socio-demographic characteristics

A total of 201 pregnant women of age ranging 17 to 35 years (mean 23.64 years) had participated in this study. About half (48.3%) of the study participants were in the age range of 20–24 years. Majority of them (80.6%) were house wives in occupation. Nearly a quarter of the pregnant women were illiterate (who were unable to read and write) (Table 1). Eighty three (41.3%) of the pregnant women were primigravidae, and the remaining were multigravidae**.**

**Table 1:** **Distribution of *T. gondii *along with demographic characteristics of the pregnant women (n = 201), Jimma town, 2011**

**Demographic characteristics**	**Seroprevalence**		**Total n (%)**
	**Positive n (%)**	**Negative n (%)**	
**Age group (years)**			
15 – 19	16 (64.0)	9 (36.0)	25 (12.4)
20 – 24	81 (83.5)	16 (16.5)	97 (48.3)
25 – 29	55 (88.7)	7 (11.3)	62 (30.8)
30 – 35	16 (94.1)	1 (5.9)	17 (8.5)
**Occupation**			
Housewives	134 (82.7)	28 (17.3)	162 (80.6)
Merchants	14 (82.4)	3 (17.6)	17 (8.5)
House maids	6 (75)	2 (25)	8 (4.0)
Daily laborers	7 (100)	0	7 (3.5)
Others	7 (100)	0	7 (3.5)
**Level of education**			
Illiterate	38 (77.6)	11 (22.4)	49 (24.4)
Read and write only	13 (86.7)	2 (13.3)	15 (7.5)
Grade 1-4	32 (82.1)	7 (17.9)	39 (19.4)
Grade 5-8	44 (86.3)	7 (13.7)	51 (25.4)
Grade 9-12	37 (90.2)	4 (9.8)	41 (20.4)
12+	4 (66.7)	2 (33.3)	6 (3.0)
**Trimester of pregnancy**			
1^st^ trimester	23 (79.3)	6 (20.7)	29 (14.4)
2^nd^ trimester	86 (82.7)	18 (17.3)	104 (51.7)
3^rd^ trimester	59 (86.8)	9 (13.2)	68 (33.8)

### Seroprevalence of *T. gondii*

The overall seroprevalence of *T. gondii* among the pregnant women was 83.6%. One hundred and sixty three (81.1%) of them were IgG seropositive, indicating either past infection or acquired immunity. Five (2.5%) of them were IgM seropositive, three of the five were positive for both IgM and IgG.

### Factors associated with *T. gondii*

Of the total 201 study participants, about 8.5% were within age range of 30–35 years. Amongst these age groups, 94.1% of them were positive for anti-*T. gondii* antibody. Anti-*Toxoplasma* seroprevalence of the pregnant women showed an increasing pattern of seropositivity with increasing age group. After adjusting for other factors, age showed significant association (p = 0.03) with *T. gondii* infection (Table 2).

**Table 2:** **Factors associated with *Toxoplasma gondii *infection among the pregnant women (n = 201) in Jimma town, 2011**

**Characteristics**	**Seroprevalence**		**COR (95%CI)**	**AOR (95%CI)**
	**Positive n(%)**	**Negative n(%)**		
**Educational status**				
Illiterate	38 (77.5)	11 (22.5)	1.7 (0.76-3.84)	0.66 (0.27-1.60)
Literate	130 (85.5)	22 (14.5)	1	1
**Occupation**				
House wives	134 (82.7)	28 (17.3)	0.7 (0.25-1.96)	0.67 (0.23-1.99)
Others	34 (87.2)	5 (12.8)	1	1
**Age group (years)**				
15 – 19	16 (64.0)	9 (36.0)	1	1
20 – 24	81 (83.5)	16 (16.5)	2.8 (1.07-7.56)*	3.18 (1.08-9.34)*
25 – 29	55 (88.7)	7 (11.3)	4.4 (1.42-13.73)*	4.95 (1.41-17.38)*
30 – 35	16 (94.1)	1 (5.9)	9.0 (1.02-79.54)*	13.98 (1.42-137.92)*
**Presence of cats**				
Yes	56 (94.9)	3 (5.1)	5.0 (1.46- 17.10)*	5.82 (1.61-20.99)*
No	112 (75.6)	30 (24.4)	1	1
**Contact with soil**				
Yes	121 (81.8)	27 (18.2)	0.57 (0.22-1.47)	0.63 (0.23-1.74)
No	47 (88.7)	6 (11.3)	1	1
**Raw meat eating habit**				
Yes	87 (82.9)	18 (17.1)	0.89 (0.42-1.89)	0.73 (0.31-1.72)
No	81 (84.4)	15 (15.6)	1	1
**Source of drinking water**				
Well	20 (80)	5 (20)	0.75 (0.26-2.18)	1.22 (0.36-4.15)
Pipe	148 (84.1)	28 (15.9)	1	1

*Statistically significant at P < 0.05, COR = Crude Odds Ratio, AOR = Adjusted Odds Ratio, CI = Confidence Interval.

With regard to the association of seroprevalence with educational background, 49 (24.4%) of the pregnant women were illiterate, of which 77.6% were seropositive. There was no significant difference (p = 0.19) in seropositivity rate between the illiterate and literate study participants. Regarding occupation of the participants, 80.6% of them were housewives. Of these, about 82.7% were seropositive for *T. gondii.* There was no significant difference (p = 0.5) in *Toxoplasma* seropositivity among individuals with different occupation. All the pregnant women responded had no history of blood transfusion. About 51.7% of the pregnant women were within their second trimester gestational period. The study showed that an increase in trimester had a corresponding increase with distribution of the infection.

Domestic cats were recorded from fifty nine (29.4%) of the study households, of which 95% were positive for anti-*T. gondii* antibody. Participants having domestic cat at home showed significant association (OR = 5.82, 95% CI: 1.61-20.99, p < 0.05) with *T. gondii* antibodies (Table 2). Over 50% of the study participants reported to have a habit of eating raw meat, of which, 82.9% were *T. gondii* seropositive. However, there was no significant association (p = 0.7) between habit of eating raw meat and *T. gondii* seropositivity. Overall, 87.6% of the study participants reported to use pipe water as a source of drinking water. Seroprevalence among those who reported to use pipe water was 84.1%. Source of drinking water did not show significant association (p = 0.6) with *Toxoplasma* seropositivity (Table 2). Majority of the pregnant women (73.6%) had reported to have a history of engagement in farming activities, which could indicate frequent contact with soil. Of these, 121 (81.8%) of them were seropositive. Having a history of contact with soil did not demonstrate significant association (P = 0.2) with *Toxoplasma* seropositivity.

## Discussion

This study showed an overall 83.6% seroprevalence of anti-*T. gondii* antibody among pregnant women in Jimma town. This finding was higher than the prevalence among the general population reported from Nazareth town, Ethiopia, in which 60% of the sampled population had evidence of *T. gondii* infection [9]. It was also higher than the seroprevalence reported before two decades on samples collected from different regions of Ethiopia [10].

Similarly, the IgG seroprevalence of *T. gondii* obtained in this study was higher than those reported from Palestine [11], Saudi Arabia [12], Brazil [13], Sudan [14], Morocco [15] and China [3]. In contrast, lower seroprevalence of *T. gondii* was reported in many European countries and the United States of America [2]. This wide variability could be attributed to differences in climatic conditions and personal hygienic practices, feeding habits, socio-economic and literacy status of the study subjects.

On the other hand *T. gondii* overall prevalence was lower than the prevalence among pregnant women in Ghana, where the seroprevalence was 92.5% [4]. The observed difference in the rates of infection could be due to variation in age distribution and antibody profiles of the study populations.

In the current study, increase in seropositivity of *antiT. gondii* antibody was observed as age increases, which is in agreement with other previous similar studies [11,16]. This could be explained by the fact that older women are more likely to have been exposed to any one of the risk factors than younger women as a result of longer exposure time.

Contact with domestic cats is often mentioned as a risk factor, however, there are also contradicting reports. Our findings showed significant association between *T. gondii* infection and presence of domestic cats at home, which was one of the predictors for *T. gondii* infection in this study. This finding corroborates with studies reported from France [17] and Taiwan [18]. In contrast, some studies reported absence of association between *Toxoplasma* infection and presence of domestic cats in the household [11,19,20]. The way the cats’ litter box is cleaned rather than the simple presence of cats could account for exposure of individuals to the parasite. Moreover, the prevalence of the parasite among the domestic cats may depend on the type of cats (stray vs pet cats) in different countries, in that stray cats were reported to be more exposed to the parasite as compared to pet cats [21]. In the present study area stray cats were more common and it was expected that the prevalence would be higher.

Contaminated drinking water is also a potential source of *T. gondii* infection [22]. A study done in Nigeria had also reported higher seroprevalence rate among pregnant women drinking well water compared to those using packed water [20]. Though, in our study, 12.4% of the study participants reported to use water from well for drinking when pipe water is interrupted or as their sole source of drinking water there was no association between source of water for drinking and *Toxoplasma* infection.

In the present study, it was observed that 105 (52.2%) of the pregnant women reported to eat raw meat but showed no significant association with *Toxoplasma* infection, which is consistent with studies from Turkey [19] and Palestine [11]. However, other studies by Elnahas *et al.*[14] and Ghoneim *et al.*[23] reported an association of raw meat consumption with *Toxoplasma* infection. This variation could be due to differences in the prevalence of the parasite in the animals in those countries as well as the type of animals consumed. In a seroepidemiological survey of toxoplasmosis conducted before two decades among domestic animals in Ethiopia, it was reported that 22.9% the sheep, 11.6% of the goats and 6.6% of the cattle examined were seropositive [24]. In Jimma town, it is more likely that raw beef is consumed more often than raw meat of goats or sheep. This indicates that raw beef may not be the major route for the transmission of the parasite in the area. However, vertical transmission may contribute for the high seroprevalence of the parasite as it was reported in a previous study [25].

## Conclusions

Seroprevalence of *T. gondii* antibodies was high among pregnant women and the prevalence showed a corresponding increase as the age of the pregnant women increases. Presence of domestic cats at homes of the pregnant women was identified to be main factor for *T. gondii* infection. Therefore, awareness creation on the modes of transmission and prevention of *T. gondii* should be made to women of child bearing age in general and pregnant women in particular during their antenatal care follow up. Moreover, there is need to control urban stray cat population to reduce the risk of zoonotic transmission of the parasite.

## Competing interests

We declare that we do not have any conflict of interests.

## Authors’ contributions

EZ conceived the study, participated in the study design, data analysis and drafted the manuscript. SA, TB, AS coordinated specimen collection and laboratory work, participated in data analysis. AZ participated in the design, supervised data collection and participated in data analysis and DY critically reviewed the manuscript. All authors read and approved the final manuscript.

## Pre-publication history

The pre-publication history for this paper can be accessed here:

http://www.biomedcentral.com/1471-2334/12/337/prepub

## Supplementary Material

Additional file 1 **(A) Questionnaire developed to assess socio-demographic characteristics of study participants.** (B). Questionnaire developed to assess risk factors associated with *Toxoplasma* infection. Click here for file
