# Supplementary material for: Seroprevalence of Toxoplasma gondii and associated risk factors among pregnant women in Jimma town, Southwestern Ethiopia
Source: BMC Infect Dis. 2012 Dec 5;12:337. doi: 10.1186/1471-2334-12-337 (PMC3519766; doi:10.1186/1471-2334-12-337)
Supplement: Additional file 1 — (A) Questionnaire developed to assess socio-demographic characteristics of study participants. (B). Questionnaire developed to assess risk factors associated with Toxoplasma infection. [file 1471-2334-12-337-S1.docx]

| **Q. no** | **Questions** | **Response** | **Code** |
| --- | --- | --- | --- |
| **A1** | How old are you now? |  |  |
| **A2** | What is your current occupation? | 1. Housewives 2. Merchants 3. House maids 4. Daily laborers 5. Others |  |
| **A3** | What was the highest level of schooling you attended? | 1. Did not attend school (Illiterate ) 2. Able to read and write but no formal education 3. Grade 1-4 4. Grade 5-8 5. Grade 9-12 6. 12+ |  |
| **A4** | What was the date of your last menstrual period? |  |  |
| **A5** | How many times have you been pregnant before? |  |  |

**Additional file 1**

**A. Questionnaire developed to assess socio-demographic characteristics of study participants**

**B. Questionnaire developed to assess risk factors associated with *Toxoplasma* infection**

| **Q. no** | **Questions** | **Response** | **Code** |
| --- | --- | --- | --- |
| **B1** | Do you have domestic cat(s) at home? | 1. Yes 2. No |  |
| **B2** | Have you been involved in farming activities/gardening? | 1. Yes 2. No |  |
| **B3** | Do you usually eat raw meat? | 1. Yes 2. No |  |
| **B4** | Where do you get your drinking water? | 1. Well 2. Pipe |  |
| **B5** | Have you ever received blood for any medical reasons? | 1. Yes 2. No |  |
